# GATA5 inhibits hepatocellular carcinoma cells malignant behaviours by blocking expression of reprogramming genes

**DOI:** 10.1111/jcmm.14144

**Published:** 2019-01-22

**Authors:** Haipeng Feng, Mingyue Zhu, Ruizhu Zhang, Qiaoyun Wang, Wei Li, Xu Dong, Yi Chen, Yan Lu, Kun Liu, Bo Lin, Junli Guo, Mengsen Li

**Affiliations:** ^1^ Hainan Provincial Key Laboratory of Carcinogenesis and Intervention Hainan Medical College Hainan Province Haikou PR. China; ^2^ Key Laboratory of Molecular Biology Hainan Medical College Hainan Province Haikou PR. China; ^3^ Institution of Tumor Hainan Medical College Hainan Province Haikou PR. China

**Keywords:** GATA5 expression, HCC, reprogramming genes, stemness marker, Wnt/β‐catenin

## Abstract

Evidence indicated that GATA5 may suppress hepatocellular carcinoma (HCC) cell malignant transformation, but the mechanism of how GATA5 affects cancer cell reprogramming to inhibit HCC malignant behaviour is still unclear. In this study, we report that the expression of β‐catenin and reprogramming genes p‐Oct4, Nanog, Klf4, c‐myc and EpCAM was significantly higher in HCC tissues compared to normal liver tissues. In contrast, the expression of GATA5 was significantly lower in HCC tissues compared to normal liver tissues. Transfection of CDH‐GATA5 vectors into HCC cells (HLE, Bel 7402 and PLC/PRF/5 cells) increased the GATA5 expression and decreased the expression of β‐catenin and reprogramming genes p‐Oct4, Nanog, Klf4, c‐myc and EpCAM. Increased GATA5 expression by transfection with its expression vectors was also able to inhibit the cell growth, colony formation and capability of migration, invasion, while promoting apoptosis in HCC cells. Results revealed that GATA5 co‐localization with β‐catenin in the cytoplasm, preventing β‐catenin from entering the nucleus. Treatment with the specific Wnt/β‐catenin pathway inhibitor salinomycin was able to reduce the expression of β‐catenin and reprogramming genes. Salinomycin exerted a similar influence as GATA5, and siRNA‐GATA5 restored β‐catenin and reprogramming gene expression. This study demonstrates that an increase in the expression of GATA5 inhibits the expression of β‐catenin and reprogramming genes and suppresses tumour growth, colony formation, metastasis and invasion, while promoting apoptosis in HCC cells. The mechanism of GATA5 inhibiting the malignant behaviours of HCC cells may involve in the disruption of the Wnt/β‐catenin pathway and the reduction of reprogramming gene expression.

## 1 INTRODUCTION

Hepatocellular carcinoma (HCC) is the most common type of liver cancer, causing approximately 1 million deaths annually.^1^ Lack of early detection and effective treatment for HCC causes the high mortality rate. Therefore, the identification of the key mechanisms and optional therapies for early‐stage diagnosis and effective treatment are of great importance.^2^, ^3^ HCC development, similar to other types of cancer, is a complicated, multi‐step process.^4^ GATA transcription factors have a critical role in suppressing the development of various types of human cancer, including HCC.^5^, ^6^, ^7^

The GATA protein family (GATA1‐6) is comprised of transcription factors that bind to the DNA consensus GATA sequence.^8^ GATA1, GATA2 and GATA3 play essential roles in regulating the transcription of genes involved in cellular lineage determination,^9^ while GATA4, GATA5 and GATA6 are involved in organs originating from the mesoderm or endoderm, such as the liver, lungs, heart and gut.^10^ However, in adults, GATA transcription factors may suppress the malignant transformation of various types of human cancer.^5^, ^6^, ^11^, ^12^ For example, the loss of GATA5 expression induces the growth and colony formation in HCC cells, and up‐regulated GATA5 inhibits cholangiocarcinoma cell (CCA) growth and metastasis.

Cancer stem cells (CSCs) are believed to play a major role in HCC initiation, therapy resistance, and ultimately, in relapse. The goal of the past decade had been to prospectively identify CSCs and to identify therapeutic strategies to safely eliminate this cell population.^13^ Recent findings reported that GATA5 suppresses HCC malignant transformation.^5^, ^6^ As such, the identification of the mechanisms whereby GATA5 inhibits cancer cell reprogramming of CSCs to suppress HCC malignant transformation is important.

In this study, the expression levels of GATA5, β‐catenin and reprogramming genes were evaluated in the human liver tissue, HCC tissue specimens and cell lines. The influence of GATA5 on the expression of β‐catenin and reprogramming genes in HCC cells in vitro was subsequently characterized. Our results highlight the important role of GATA5 in inhibiting malignant behaviours of HCC cells through suppression of the Wnt/β‐catenin signalling pathway, resulting in the reduced expression of reprogramming genes.

## 2 MATERIALS AND METHODS

### 2.1 Patients and specimens

Archived clinical specimens were originally collected during hepatectomy of 35 patients, including eight cases of liver trauma (normal liver specimens) and 27 HCC cases at Hainan Provincial People’s Hospital (Haikou, Hainan, China) and the Affiliated Hospital of the Hainan Medical College (Haikou, Hainan, China) between January 2010 and December 2017. Of the 35 patients, 24 were men and 11 were women, with an average age of 52.3 (range, 32‐78) years. All enrolled patients were treated with radical surgery and received no other treatments. Clinical data were obtained from retrospective chart review, and follow‐up care was available for all patients. A section of liver tissue approximately 2.0 × 2.0 × 2.0 cm in size was obtained from each patient immediately after surgery. Approximately 1.0 × 1.0 × 1.0 cm of each sample was fixed in 10% formalin, embedded in paraffin and routinely stained with haematoxylin and eosin. The 1.0 × 1.0 × 1.0 cm tissue specimens were stored in liquid nitrogen. All specimens were assessed blindly and independently by two pathologists. In the case of interobserver disagreement, final decisions were achieved by general consensus. All selected patients were diagnosed by histopathological evaluation and computerized tomography (CT). The study protocol was approved by the Ethics Committee of Hainan Provincial People’s Hospital and the Science Investigation Ethics Committee of Hainan Medical College. Written informed consent was obtained from all participants. Methods were performed in accordance with the approved guidelines.^14^

### 2.2 Immunohistochemical analysis

The expression and cellular distribution of GATA5, β‐catenin, p‐Oct4 (Thr235), Nanog, Klf4, c‐myc and EpCAM proteins in HCC specimens were assessed by immunohistochemical analysis. Five‐millimetre‐thick paraffin sections were deparaffinized and rehydrated according to standard protocols, and heat‐induced antigen retrieval was performed in a sodium citrate buffer (10 mmol/L, pH 6.0). Endogenous peroxidase was inhibited by 0.3% H_2_O_2_, and non‐specific protein binding was blocked with 10% goat serum. The sections were then incubated with primary antibody against GATA5, β‐catenin, p‐Oct4 (Thr235), Nanog, Klf4, c‐myc and EpCAM (1:100 dilution; Santa Cruz Biotechnology Inc, Santa Cruz, CA, USA) at 4°C overnight. Non‐immune immunoglobulin G (IgG) was used as a negative control, and antigenic sites were localized using an SP9000 Polymer Detection System and a 3,3′‐diaminobenzidine kit (ZSGB‐BIO, Beijing, China). Methods were performed in accordance with the approved guidelines.^14^

### 2.3 Cell culture

The HCC cell lines HLE, Bel 7402 and PLC/PRF/5 were gifts from the Department of Cell Biology, Peking University Health Science Center (Beijing, China) and were cultured in DMEM medium containing 10% imported fetal bovine serum, 100 units/mL of penicillin in a cell culture incubator at 37°C and 5% CO_2_ saturated humidity, according to the growth state of the cells and was changed every 1‐2 days.^15^

### 2.4 Construction of GATA5 expression vector and transfection

pcDNA3.1‐GATA5 was constructed as follows. Full‐length human GATA5 cDNA (residue 1‐397, NCBI: NM_080473) was synthesized with a Kozak sequence GCCACC at the N‐terminus. Then, the synthetic cDNA was amplified by PCR using the following primer pairs: 5′CCGAAGCTTGCCACCATGTACCAGAGCCT‐3′ and 5′‐CGGGCGGCCGCCTAGGCCAAGGCCAGCGC‐3′. The *Hin*dIII and *Not*I restriction sites are underlined. The PCR product was digested with *Hin*dIII and *Not*I restriction enzymes (Takara Bio Inc, China) and ligated into the expression vector pcDNA3.1(+) (Invitrogen, USA). The stable expression vector CDH‐GATA5 construct was similar to the pcDNA3.1‐GATA5. The difference between them was that the CDH‐GATA5 vector colony was cloned into the pCDH‐CMV‐MCS‐EF1‐coGFP plasmid (SystemBio, USA) using restriction enzymes *Bam*HI/*Eco*RI (Takara Bio Inc, China). The expression vector was transformed into *Escherichia coli* and used for amplification.

The transfection of GATA5 expression vectors into HCC cells was induced by Lipofectamine 2000 (Invitrogen). For stable expression vectors CDH‐GATA5, 400 mg/mL G418 was applied to screen stable cell clones, and the transfection of HLE, Bel7402 and PLC/PRF/5 cells was termed HLE‐GATA5, Bel7402‐GATA5 and PLC/PRF/5‐GATA5.

### 2.5 RNA interference

For the RNA interference (RNAi) experiments, siRNA‐GATA5 was applied to inhibit GATA5 expression. Operation steps were as follows. HLE, Bel7402 and PLC/PRF/5 cells were seeded into six‐well plates and cultured until they reached 80%‐90% confluence. Then, transfection of siRNA‐GATA5 or its negative control was performed in each well in the absence of serum. The transfection of siRNA‐GATA5 vectors into the cells were induced by Lipofectamine 2000 (Invitrogen). The siRNA sequence is as follows: 5′‐AAAGUCCUCAGGCUCGAAC‐3′.

### 2.6 Semi‐quantitative reverse transcription‐polymerase chain reaction analysis

GATA5 RNA and cDNA were prepared by the manufacturer’s recommended protocol using reverse transcriptase and random hexamers from a RevertAid First Strand cDNA Synthesis Kit (Fermentas). The previously reported primers used for quantifying GATA5 mRNA expression were synthesized by TaKaRa (Dalian, China). The primers of GATA5 were as follows: Sense, 5′TCGCCAGCACTGACAGCTCAG‐3′ and antisense, 5′‐TGGTCTGTTCCA GGCTGTTCC‐3′. The primers of GAPDH were as follows: Sense, 5′‐AAA TCC CAT CAC CAT CTT CCA G‐3′ and antisense, 5′‐TGA GTC CTT CCA CGA TAC CAA A‐3′. The PCR reaction was also performed with rTaq (TaKaRa) in a DNA thermal cycler (Maxygen) according to a standard protocol as reported in a described previously.^16^

### 2.7 Western blotting and co‐immunoprecipitation analysis

The cultured cells were collected and lysed using cell lysate to collect the proteins. The target proteins were isolated by SDS‐PAGE gel electrophoresis. After protein transfer, the milk was blocked, and the following primary antibodies (all from Santa Cruz Biotechnology Inc): rabbit anti‐GATA5 (1:1000), rabbit anti‐EpCAM (1:1000), rabbit anti‐KLF4 (1:1000), rabbit anti‐p‐Oct4 (1:1000), mouse anti‐c‐myc (1:1000), rabbit anti‐Nanog (1:1000), mouse anti‐β‐catenin (1:1000) were added to the membranes and incubated overnight at 4°C. After three washes with TBST, the membranes were incubated with horseradish peroxidase‐conjugated secondary antibodies for 1 hour at 37°C. The bands were visualized using enhanced chemiluminescence reagents (Thermo Fisher, Rockford, IL, USA) and analysed with a gel analysis system (VersDoc TM5000MP System; Bio‐Rad, Guangzhou, China). The expression of GAPDH was used as a loading control.^16^ Co‐immunoprecipitation (Co‐IP) was employed to assess the binding of GATA5 to β‐catenin in cell lines, the method as described previously.^17^

### 2.8 MTT assay

Cells were digested with trypsin and diluted in DMEM containing 10% fetal bovine serum in a suspension of 2.5 × 10^4^ cells/mL, and 200 μL/well was subcultured in 96‐well plates. After incubation for 72 hours in the well plates, a MTT solution (5 mg/mL) was added to each well of the cells, and the culture was continued for 4 hours. The culture medium containing MTT was discarded, and 200 μL of dimethyl sulphoxide was added to each well. The plates were oscillated for 10 minutes. Absorbance values of the experimental group were measured by a microplate reader (Bio‐Rad) at a wavelength of 490 nm, and the growth rate was measured by MTT.^18^

### 2.9 Soft agar colony formation assay

Soft agar formation assays were performed to compare the clonogenic potential of HLE, Bel7402 and PLC/PRF/5 cells while transfected with CDH‐GATA5 expressed vectors. HLE, Bel7402 and PLC/PRF/5 cells or the cells were transfected with CDH‐GATA5 expressed vectors or siRNA‐GATA5 vectors. These cells were seeded in semisolid medium. Briefly, 5000 cells were mixed with 0.5% soft agar and plated on a layer of 0.8% bottom agar in six‐well plates. A total of 2 mL complete medium was added to the top of the agar. Cells were fed twice a week, and the plates were incubated for 14 days at 37ºC with 5% CO_2_. Colonies were photographed and counted with a Nikon inverted microscope (Nikon Corp., Tokyo, Japan).^14^

### 2.10 Scratch test

Cell motility was analysed by a wound healing assay. One day before scratching, HLE, Bel7402, PLC\PRF\5 cells were transfected with CDH‐GATA5 vectors, siRNA‐GATA5 or no transfect (control cells). Cells were seeded into 12‐well plates for almost total confluence at 24 hours. A scratching wound was created by scraping the middle of the cell monolayer with a sterile micropipette tip. After all detached cells were washed away with PBS, the cells were cultured with medium containing 10% FCS, and images of cell migration into the wound area were captured at 0 and 48 hours by an inverted microscope (100×) to record the distance migrated.^15^

### 2.11 Cell migration assays

Cell migration and invasion assays were performed according to the manufacturer’s protocols. To measure cell migration, transwell chambers were used to observe cultured cell inserts (Transwell chamber; 8‐mm pore size; Costar, High Wycombe, UK). Cells were placed into the wells of 12‐well cultured plates, and the upper and lower chambers were separated. Cells (5 × 10^5^ cells/mL) were added to the upper chamber and cultured with serum‐free DMEM medium, whereas the lower chamber was filled with complete medium (containing 20% FCS). After 48 hours of incubation, the cells in the upper chamber were carefully removed with a cotton swab, and those that had migrated through the membrane to the lower surface were fixed with 90% methanol and stained with 0.1% crystal violet. The number of cells that had migrated through the pores was quantified by counting five independent visual fields under the microscope (Olympus) using a 200× objective. For invasion assays, transwell chambers were covered with Matrigel (BD Falcon, NJ, USA). The experimental procedure is similar to that for the migration assays. Three independent assays were performed.^14^

### 2.12 Flow cytometry analysis

HLE, Bel7402 and PLC/PRF/5 cells were cultured in RPMI‐1640 medium supplemented with 10% FCS at 37°C in a humidified atmosphere containing 5% CO2. The CDH‐GATA5 vector was transfected into the cells using Lipofectamine 2000 (Invitrogen) for 24 hours, then treated with paclitaxel (20 μg/mL) for 48 hours, harvested by trypsinization, washed with PBS, resuspended and incubated in PI/Annexin‐V solution (KeyGEN Biotech) for apoptosis analysis. At least 10 000 live cells were analysed on a FACSCalibur flow cytometer. Data were analysed using FlowJo7.6.1 software.^18^

### 2.13 Protein localisation analysis by laser confocal microscopy

Cells were diluted with DMEM containing 10% fetal bovine serum into a suspension of 2 × 10^4^ cells/mL cells, and 300 µL was subcultured into a chamber slide. When the cells reached 60% confluence, they were fixed and transparent. After cells were blocked, the following primary antibodies were applied: rabbit anti‐GATA5 (1:100) and mouse anti‐β‐catenin (1:100) (primary antibodies from Abcam). Samples were shaken at 4°C for 24 hours, at which time the primary antibodies were removed and PBS was applied. The cells were washed three times (5 minutes each), and Alexa Fluor 488^®^‐labelled goat anti‐rabbit or Alexa Fluor 647^®^‐labelled goat anti‐mouse secondary antibodies were added and shaken at room temperature for 2 hours (protected from light). Cells were then covered with DAPI at a concentration of 100 mg/L, shaken gently for 5 minutes at room temperature (protected from light), and washed three times with 10% PBS (protected from light). The labelling results were observed with an Olympus FluoView FV1000 (Japan) laser confocal microscope and images were obtained.^17^

### 2.14 Statistical analysis

The results of multiple observations are presented as the mean ± SD of at least three independent experiments. Statistical significance was determined using Student’s *t* test and one‐way ANOVA (SPSS 11.5 software for Windows; SPSS Inc, Chicago, IL, USA).

## 3 RESULTS

### 3.1 GATA5 reduces the expression of β‐catenin and reprogramming genes in HCC tissues and HCC cells

**Figure 1:**
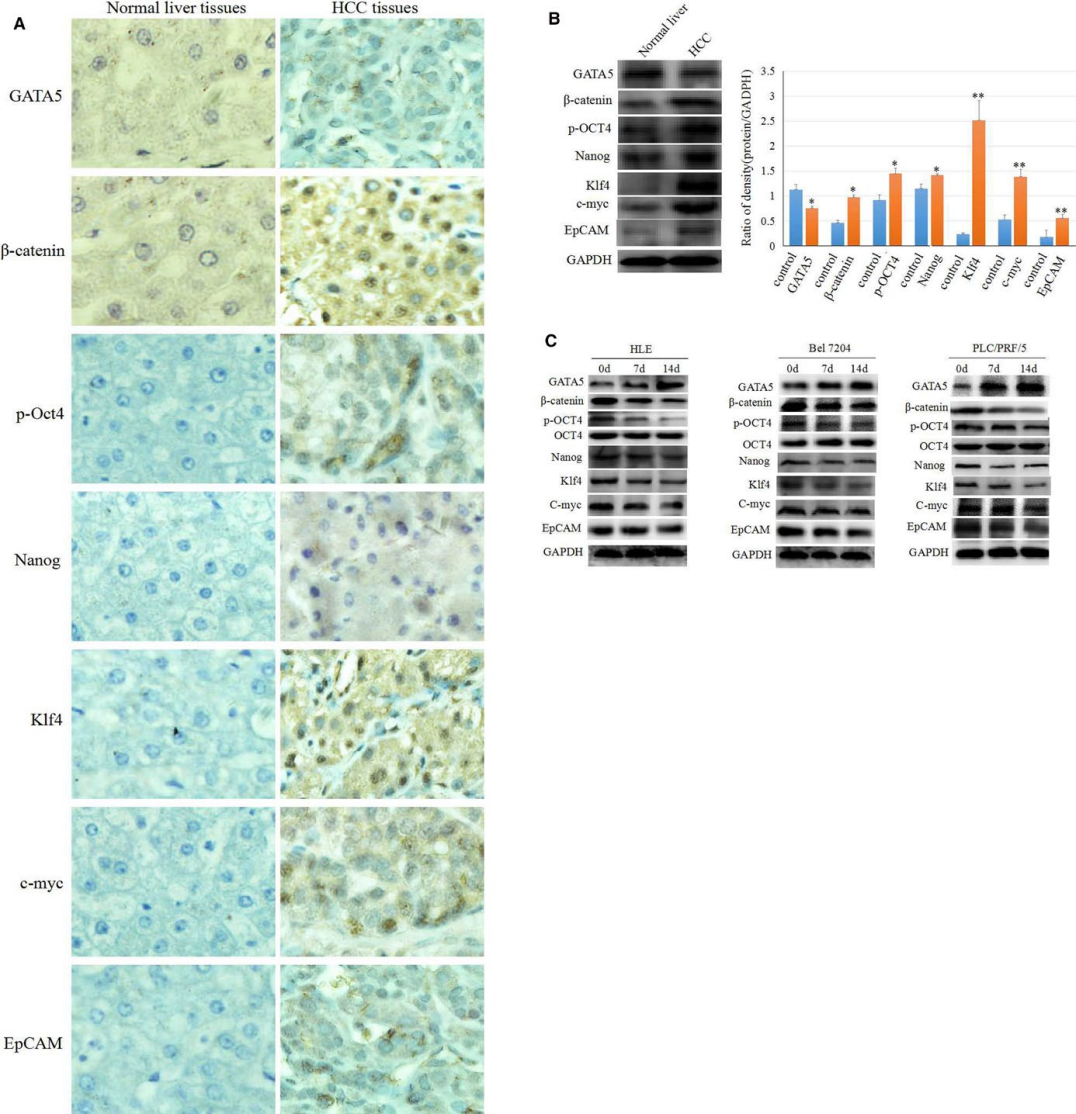
The expression of GATA5, β‐catenin and reprogramming genes in human HCC tissues and cells. A, Immunohistochemistry analysis of the expression of β‐catenin and reprogramming genes p‐Oct4, Nanog, Klf4, c‐myc and EpCAM in HCC tissues and normal human liver tissues. Brown staining represents positive expression. B, Western blot analysis of the expression of β‐catenin and reprogramming genes p‐Oct4, Nanog, Klf4, c‐myc and EpCAM in HCC tissues and normal human liver tissues, the right image indicated the expression difference of proteins, *P < 0.05, **P < 0.01 vs control groups, N = 3. C, HLE, Bel 7402 and PLC/PRF/5 cell were transfected with CDH‐GATA5 for 0, 7 and 14 d, and Western blot analysis was performed for the expression of β‐catenin and reprogramming genes p‐Oct4, Nanog, Klf4, c‐myc and EpCAM. Images are representative of three independent experiments

To observe whether GATA5 correlates with the expression of β‐catenin and reprogramming genes in HCC tissues, we performed immunohistochemical and Western blotting analysis of these proteins in normal liver tissue cases (liver trauma) and clinical HCC patient samples. The results indicated that clinical HCC tissues expressed lower levels of GATA5 but higher levels of β‐catenin and reprogramming genes p‐Oct4, Nanog, Klf4, c‐myc and EpCAM than normal liver tissues (Figure 1A,B). To further analyse whether GATA5 influences the expression of β‐catenin and reprogramming genes in HCC, we transfected HCC cells with a CDH‐GATA5 vectors, and Western blotting assays revealed that enhancing the expression of GATA5 reduced the expression of β‐catenin and reprogramming genes p‐Oct4, Nanog, Klf4, c‐myc and EpCAM (Figure 1C). These results indicated that GATA5 harbours a trait to inhibit the expression of β‐catenin proteins and reprogramming genes in HCC.

### 3.2 Enhancing GATA5 expression inhibits growth and colony formation in HCC cells

**Figure 2:**
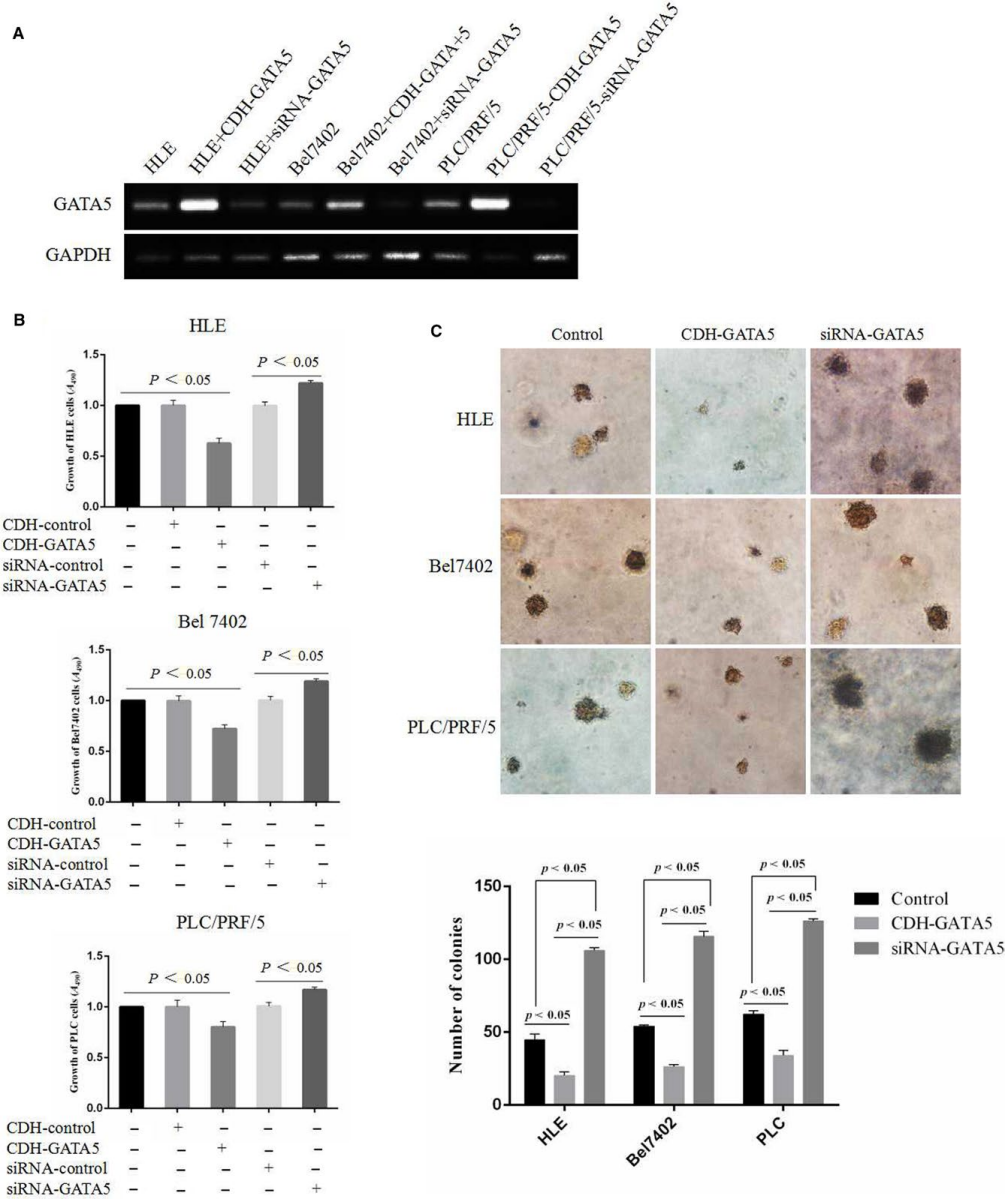
Enhancing GATA5 expression inhibits growth and colony formation in HCC cells. A, Semi‐quantitative reverse transcription‐polymerase chain reaction (RT‐PCR) was applied to analyse the effect of the expression of mRNA of GATA5 while transfected with siRNA‐GATA5 vectors in HCC cells. B, HLE, Bel 7402 and PLC/PRF/5 cell were transfected with CDH empty vector, CDH‑GATA5, siRNA‐GATA5 or siRNA‐scramble for 72 h, and the growth of the cells was measured by MTT assays. C, HLE, Bel 7402 and PLC/PRF/5 cell were transfected with CDH‐GATA5, siRNA‐GATA5 or no transfect (control cells) for 72 h, and colony formation was observed by optical microscopy (100×). *P* < 0.05 indicates statistical significance

Since GATA5 was able to reduce the expression of β‐catenin and reprogramming genes in HCC tissues and cell lines, we have been suggested that enhancing GATA5 expression by transfecting a GATA5 vector may suppress the growth and development of HCC. Reverse transcription‐polymerase chain reaction (RT‐PCR) results indicated that the HCC cells were transfected with GATA5 expressed vectors or siRNA vectors, the expression of GATA5 mRNA was significantly enhanced or inhibited (Figure 2A). Therefore, GATA5 expression vectors or siRNA vectors were transfected into HLE, Bel 7402 and PLC/PRF/5 cells. The results of the present study revealed that increasing expression of GATA5 significantly inhibited HCC cell growth compared to the cells transfected with empty vector (CDH) and the control cells (no transfection). Similarly, siRNA‐GATA5 restored HCC cell growth that was inhibited by GATA5 in these cells (Figure 2B). In addition, enhancing GATA5 gene expression also inhibited the colony formation ability of HLE, Bel 7402 and PLC/PRF/5 cells compared to the control cells, while siRNA‐GATA5 abrogated this effect (Figure 2C).

### 3.3 Enhancing GATA5 expression suppresses HCC cell scratch repair and migration

**Figure 3:**
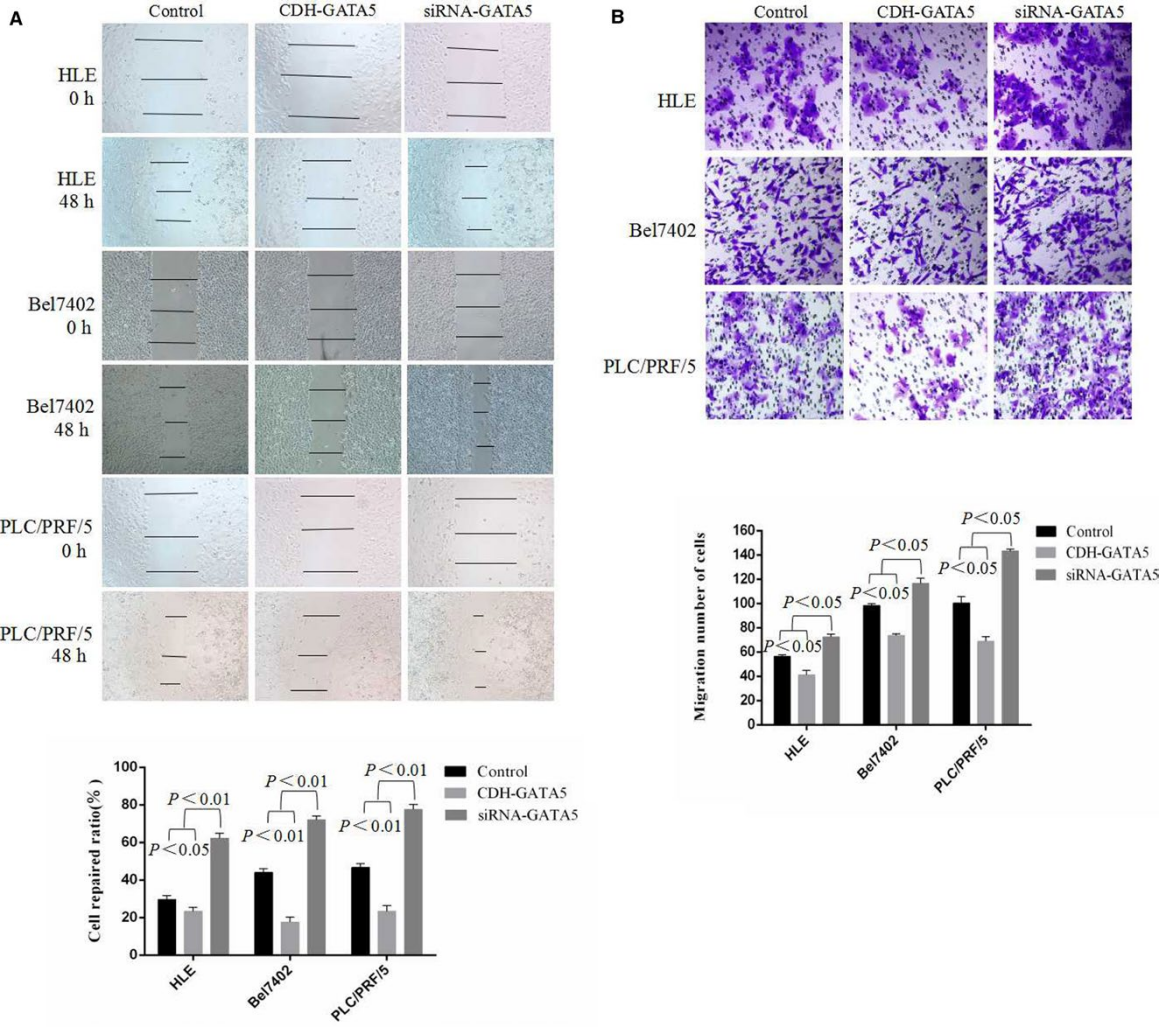
Influence of GATA5 on scratch repair and migration in HCC cells. A, HLE, Bel 7402 and PLC/PRF/5 cells were transfected with CDH empty vector, CDH‐GATA5, siRNA‐GATA5 or siRNA‐scramble for 48 h. Scratch repair was observed by microscopy. The low columnar graph shows repair ratio of the cells, *P* < 0.05 indicates statistical significance. B, HLE, Bel 7402 and PLC/PRF/5 cells were transfected with CDH‐GATA5, siRNA‐GATA5 or no transfect (control cell) for 72 h. Migratory cells were stained with 0.1% crystal violet and observed by microscopy. The low columnar graph indicates the quantity of migratory cells. *P* < 0.05 indicates statistical significance

To further demonstrate that enhancing GATA5 expression suppresses the migration of HCC cells, the effects of GATA5 on scratch repair and the migration of human liver cancer cells were observed. Scratch repair assays indicated that repair‐induced migration of HCC cells was significantly decreased, while HCC cells that were transfected with GATA5 expressed vectors (CDH‐GATA5) for 48 hours. HCC cells covered less than 40%‐60% of the scratch area during the 48 hours compared to the control cells, while transfection of siRNA‐GATA5 enhanced the scratch repair capacity of the cells (Figure 3A). These results indicated that GATA5 harbours a function to inhibit scratch repair of HCC cells in vitro. Migration assays indicated that pore transfer capacity of HCC cells was significantly decreased in cells transfected with CDH‐GATA5 vectors compared to control cells. However, the pore migration capacity of HCC cells was enhanced in response to transfection with siRNA‐GATA5 (Figure 3B). These results revealed that GATA5 plays an inhibitory role in the migration of HCC cells.

### 3.4 GATA5 inhibits the expression of MMP2 and MMP9, invasion of in HCC cells

**Figure 4:**
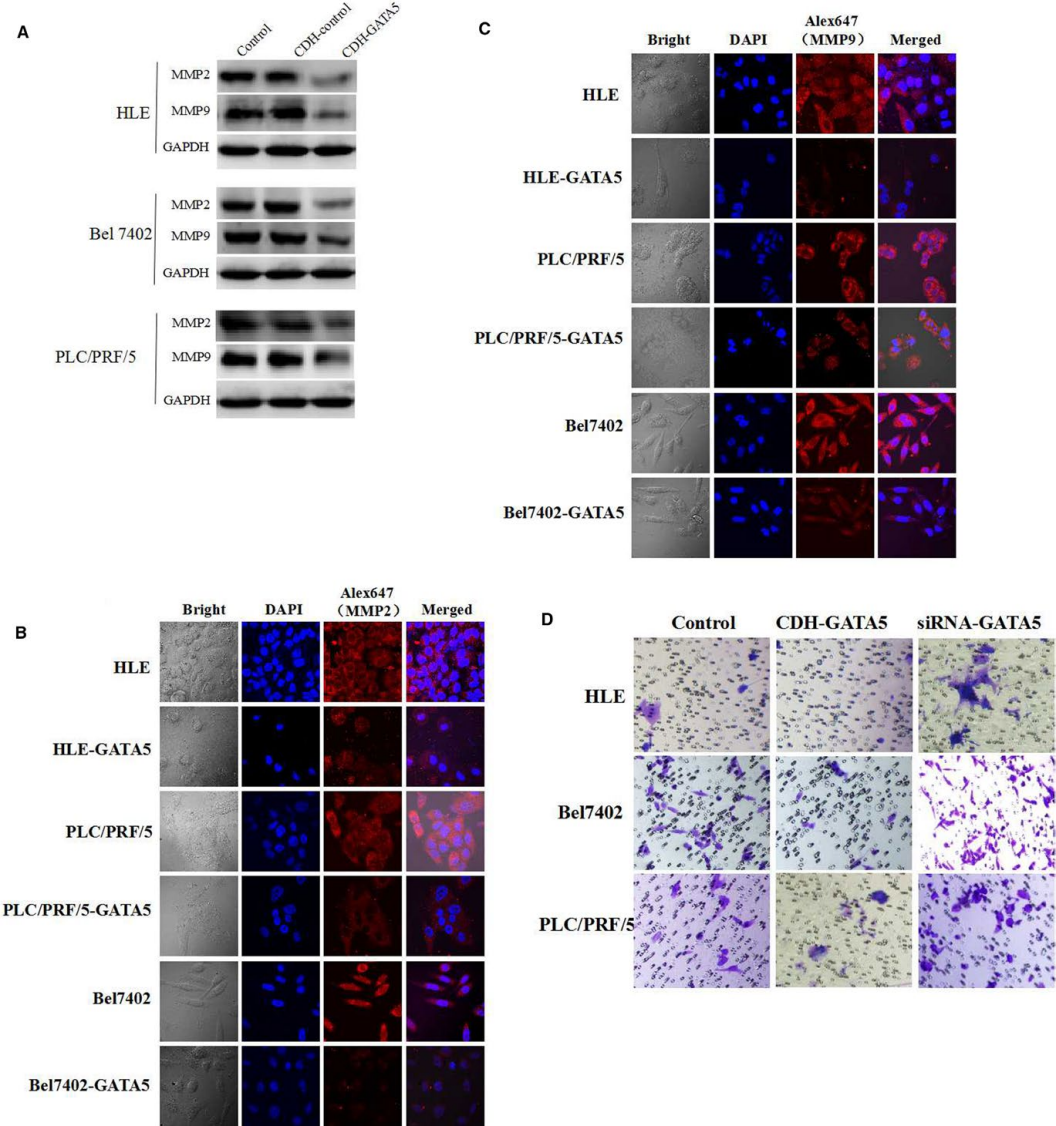
Influence of GATA5 on the expression of MMP2 and MMP9 in HCC cells. A, HLE, Bel 7402 and PLC/PRF/5 cells were transfected with CDH empty vector, CDH‐GATA5 or no transfect (control cells) for 48 h. Western blot analysis of the expression of MMP2 and MMP9 in HLE, Bel 7402 and PLC/PRF/5 cells. B and C, HLE, Bel 7402 and PLC/PRF/5 cell were transfected with pcDNA3.1‐GATA5 or no transfect (control cells) and were then cultured at 37°C in a humidified atmosphere of 5% CO_2_. Localization of MMP2 and MMP9 was visualized, and images were captured under laser confocal microscopy. Nuclei are stained with DAPI (blue). MMP2 and MMP9 are labelled with FRITC (red). D, The invasion of the cells was detected while transfected with GATA5 expressed vectors or siRNA vectors in HCC cells. Three independent experiments were performed for these data

We next assessed the influence of GATA5 on expression of the invasion‐related factors MMP2 and MMP9.^19^ In the present study, the overexpression vector CHD‐GATA5 was transfected into HCC cells, and Western blotting results indicated that overexpression of GATA5 inhibited the expression of MMP2 and MMP9 in HCC cells (Figure 4A). Laser confocal microscopy results also revealed that up‐regulated expression of GATA5 inhibits the expression of MMP2 (Figure 4B) and MMP9 (Figure 4C), inhibited the expression of GATA5 maybe promote the invasion of HCC cells (Figure 4D). These results demonstrated that GATA5 plays a role in down‐regulating the expression of metastasis‐related factors MMP2 and MMP9, and suppressing invasion of HCC cells.

### 3.5 GATA5 synergises with paclitaxel to inhibit growth and induce apoptosis in HCC cells

**Figure 5:**
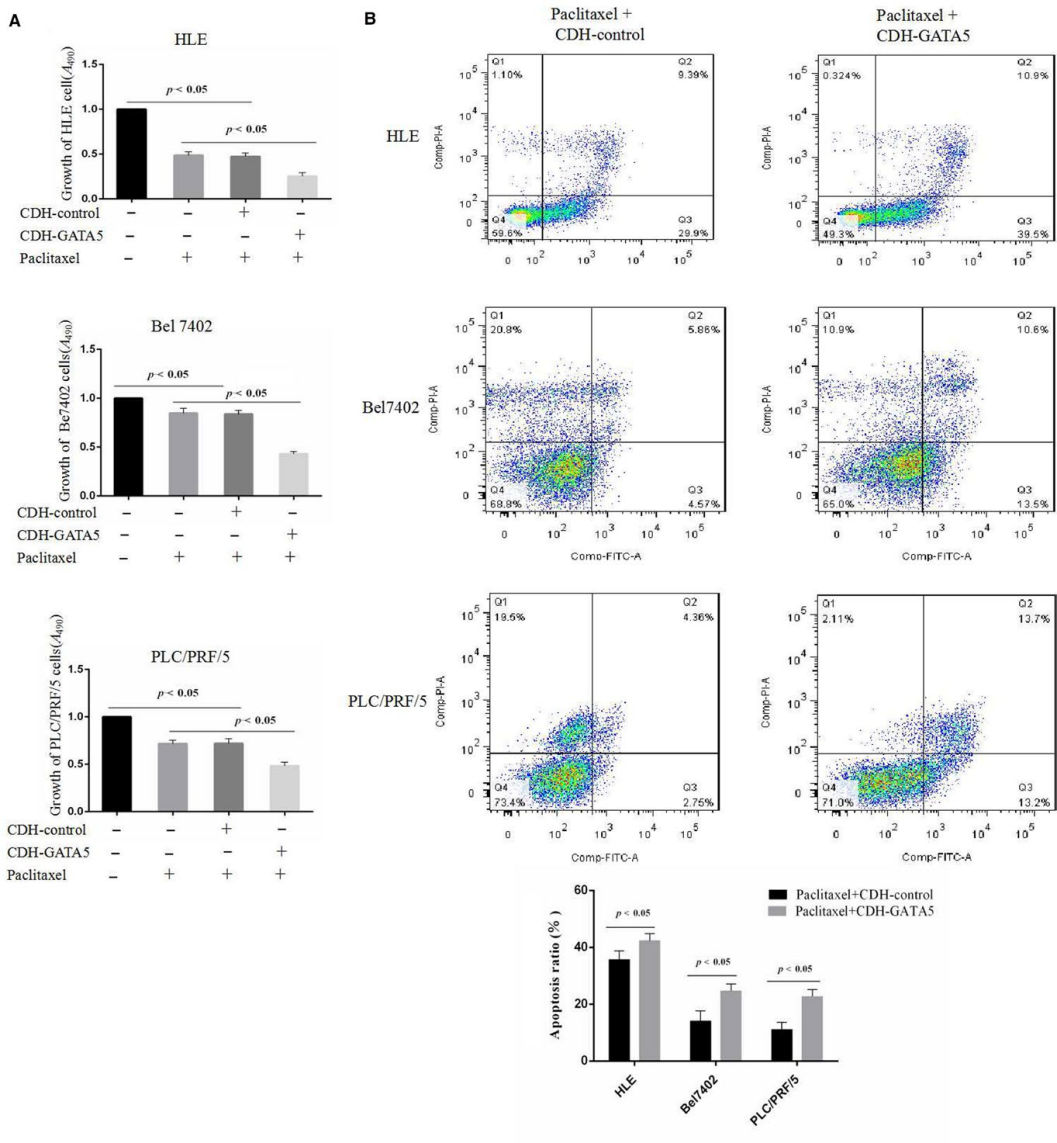
Influence of GATA5 and paclitaxel on the growth and apoptosis in HCC cells. A, HLE, Bel 7402 and PLC/PRF/5 cells were transfected with CDH empty vector and CDH‐GATA5 followed by treatment with paclitaxel (20 μg/mL) for 24 h. HCC growth was assessed by MTT *P* < 0.05 indicates statistical significance, N = 6. B, HLE, Bel 7402 and PLC/PRF/5 cells were transfected with CDH empty vector and CDH‐GATA5 followed by treatment with paclitaxel (20 μg/mL) for 24 h. Apoptosis in HLE cells was assessed by flow cytometry. The low columnar picture is the statistical analysis of the apoptosis ratios. *P* < 0.05 indicates statistical significance

To demonstrate whether GATA5 increases the sensitivity of HCC cells to pharmacological treatment, MTT was applied to analyse whether GATA5 synergism with paclitaxel inhibits the proliferation of HCC cells. HLE, Bel 7402 and PLC/PRF/5 cells were transfected with CDH‐GATA5 or a CDH empty vector followed by treatment with paclitaxel (20 μg/mL). MTT analysis indicated that HCC cell sensitivity to paclitaxel was enhanced in the presence of CDH‐GATA5 vectors for 24 hours (Figure 5A). The results showed that GATA5 synergism with paclitaxel inhibited the proliferation of HCC cells. We also applied flow cytometry analysis to examine whether GATA5 synergism with paclitaxel induced apoptosis in HCC cells. The results revealed that the number of apoptotic cells was significantly increased in HCC cells in response to transfection with CDH‐GATA5 vectors followed by paclitaxel treatment (20 μg/mL) for 24 hours (Figure 5B), confirming that GATA5 synergism with paclitaxel also enhances apoptosis in HCC cells.

### 3.6 GATA5 inhibits expression of β‐catenin and also interacts with β‐catenin in HCC cells

**Figure 6:**
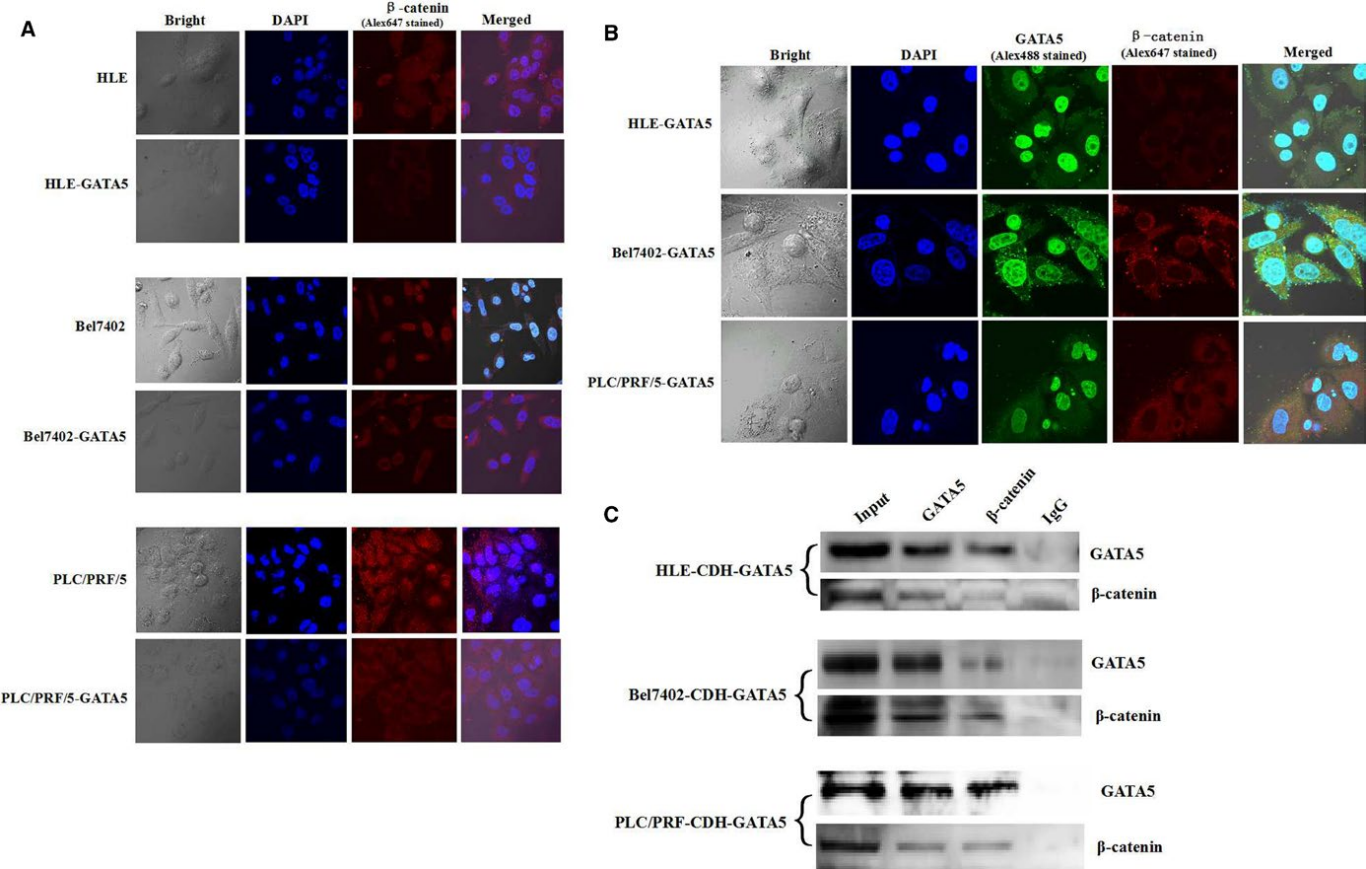
Localization of β‐catenin and co‐localization of GATA5 and β‐catenin in HCC cells. A, HLE, Bel 7402 and PLC/PRF/5 cells were transfected with pcDNA3.1‐GATA5 or no transfect (control cells), followed by culturing at 37°C in a humidified atmosphere of 5% CO_2_. Localization of β‐catenin was visualized, and images were captured under laser confocal microscopy. Nuclei are stained with DAPI (blue). β‐Catenin is labelled with FRITC (red). B, HCC cell lines transfected with pcDNA3.1‐GATA5 were cultured at 37°C in a humidified atmosphere of 5% CO_2_. Localization of GATA5 and β‐catenin was visualized. Images were captured under laser confocal microscopy. Nuclei are stained with DAPI (blue). GATA5 and β‐catenin are labelled with Alex488 (green) and Alex647 (red) respectively. C, Co‐IP was applied to analyse GATA5 interact with β‐catenin in HCC cells. Three independent experiments were performed for these data

We next investigated whether GATA5 affects Wnt/β‐catenin signalling in HCC cells. The laser confocal microscopy results indicated that the expression of β‐catenin was significantly decreased in HCC cells transfected with CDH‐GATA5. The localization results also revealed that GATA5 prevents β‐catenin from entering the nucleus (Figure 6A). The co‐localization results revealed GATA5 interaction with β‐catenin (Figure 6B), co‐IP result also indicated that GATA5 interacted with β‐catenin in HCC cells (Figure 6C). These results proved that GATA5 not only reduces the expression of β‐catenin in HCC but also interacts with β‐catenin and blocks it from entering the nucleus.

### 3.7 Silencing GATA5 expression stimulates the expression of β‐catenin and reprogramming genes in HCC cells

**Figure 7:**
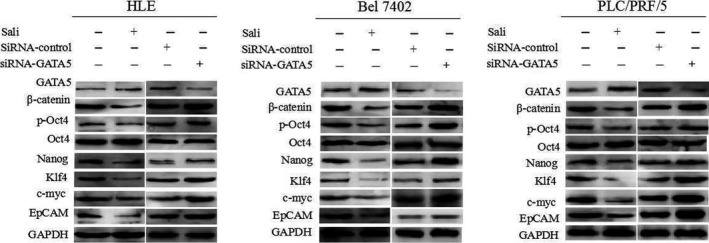
Influence of GATA5 on the expression of β‐catenin and reprogramming genes in HCC cells. HLE, Bel 7402 and PLC/PRF/5 cells were transfected with CDH‐GATA5, siRNA‐GATA5 or were treated with salinomycin (Sali) for 72 h followed by Western blot analysis of the expression of GATA5, β‐catenin, and reprogramming genes p‐Oct4, Nanog, Klf4, c‐myc and EpCAM. Images are representative of three independent experiments

We further analysed the effects of GATA5 on the β‐catenin signalling pathway transduction in HCC cells using siRNA‐GATA5 and the signalling pathway inhibitor salinomycin (Sali). Western blot results indicated that the signalling pathway inhibitor Sali, which is functionally similar to GATA5, repressed the expression of β‐catenin and reprogramming genes. Expression of β‐catenin was enhanced in HCC cells treated with siRNA‐GATA5 (Figure 7). Thus, GATA5 suppresses HCC malignant transformation through disruption of the Wnt/β‐catenin signalling pathway and reduces the expression of reprogramming genes in HCC.

## 4 DISCUSSION

Cancer stem cells play a central role in carcinogenesis and are thought to be responsible for tumour initiation, progression and relapse, as well as metastasis and drug resistance.^20^, ^21^, ^22^ CSCs contribute to cancer aggressiveness and are related to poor outcome.^23^ Reprogramming genes, such as the transcription factors p‐Oct4, Klf4, Nanog and c‐myc, play pivotal roles in reprogramming cancer cells to confer CSCs properties and neoplastic transformation in downstream progeny.^22^ EpCAM (epithelial cell adhesion molecule) is involved in epithelial‐mesenchymal‐transition, which causes cancer cells to lose epithelial polarity, resulting in increased migratory and tumour initiating potential.^23^ EpCAM was first found to contribute to homotypic cell adhesion, but it can also reprogram cancer cells. Cleavage of EpCAM causes release of its intracellular domain into the nucleus, where it forms a complex with β‐catenin and other transcription factor to regulate the transcription of various genes, some of which cause malignant transformation of cancer cells.^24^ In our study, we performed immunohistochemical analysis and examined the expression of GATA5 and reprogramming genes in HCC tissue samples. The results demonstrated that HCC tissues express higher levels of reprogramming genes and low levels of GATA5. Increasing GATA5 expression levels in HCC cells transfected with a GATA5 expression vectors reduced the expression levels of reprogramming genes. These results suggested that GATA5 may inhibit HCC development through the suppression of reprogramming gene expression.

To further demonstrate that enhancing the expression of GATA5 by expression vectors could suppress HCC cell proliferation, we transfected GATA5 expression vectors into the human liver cancer cell lines HLE, Bel 7402 and PLC/PRF/5. The result indicated that increasing expression of GATA5 inhibits both growth and colony formation. In previous studies, the reduction of GATA4 and GATA5 expression in various human cancers, including HCC, was shown to be due to gene promoter methylation in various human cancers, including HCC. Treatment with a demethylating agent restored GATA4 and GATA5 expression, inhibiting colony formation and inducing apoptosis of HCC cells in vitro.^5^ In the present study, we demonstrated that enhancing expression of GATA5 by transfection with GATA5 expression vectors also inhibits growth and colony formation of HCC cells. We also performed scratch and cell migration assays to further demonstrate that increasing GATA5 expression suppresses HCC malignant transformation. The scratch assay indicated that enhancing the expression of GATA5 in HCC cells significantly inhibits reparation in the scratch area. The cell migration assay indicated that increasing the expression of GATA5 significantly reduces the pore migratory capacity of HCC cells. Furthermore, GATA5 synergizes with paclitaxel to inhibit the growth and stimulate the apoptosis in HCC cells. These results further demonstrated that enhancing the expression of GATA5 in HCC inhibits the migration and invasion, while increasing the cells’ sensitivity to drugs.

The Wnt signalling cascade is associated with many malignancies. It contributes to carcinogenesis by regulating the expression of large numbers of genes in tumour cells.^25^ In canonical Wnt‐signalling, β‐catenin is inhibited and accumulates in the cytoplasm, where it then it translocates to the nucleus to interact with transcription activators and drive the expression of Wnt target genes.^22^, ^26^, ^27^ This study also revealed that GATA5 interacts with β‐catenin in the cytoplasm, inhibiting β‐catenin from entering the nucleus. These results demonstrated that GATA5 suppresses HCC malignant behaviour by inhibiting β‐catenin translocation to the nucleus and the subsequent expression of Wnt target genes.

The Wnt‐signalling pathway has emerged as a pivotal player in the reprogramming of cancer cells for invasion, migration and metastases in human cancer.^28^, ^29^ Reprogramming genes include p‐Oct4, Nanog, Klf4, c‐myc and EpCAM, which encourage stem cell replication that may be regulated by the Wnt‐signalling pathway.^22^, ^30^ However, the mechanism of reprogramming genes regulated by the Wnt‐signalling pathway in HCC remains unclear. In this study, we found that GATA5 reduces the expression of β‐catenin and these reprogramming genes in HCC cells. Accordingly, we also observed that GATA5 inhibits β‐catenin from entering the nucleus to induce the expression of Wnt target genes, further suggesting that these reprogramming genes belong to the Wnt‐signalling pathway downstream effectors regulated by Wnt/β‐catenin. A previous study found that GATA4 and GATA5 might be involved in the regulation of the Wnt signalling pathway in HepG2 cells through Luciferase reporter assay.^5^ In this study, we found that the expression of β‐catenin and reprogramming genes was down‐regulated by GATA5, and GATA5 colocalizes with β‐catenin in the cytoplasm, inhibiting β‐catenin entry into the nucleus. This further demonstrated that GATA5 suppresses malignant behaviour of HCC cells through deactivating Wnt/β‐catenin signalling and inhibiting the expression of reprogramming genes.

**Figure 8:**
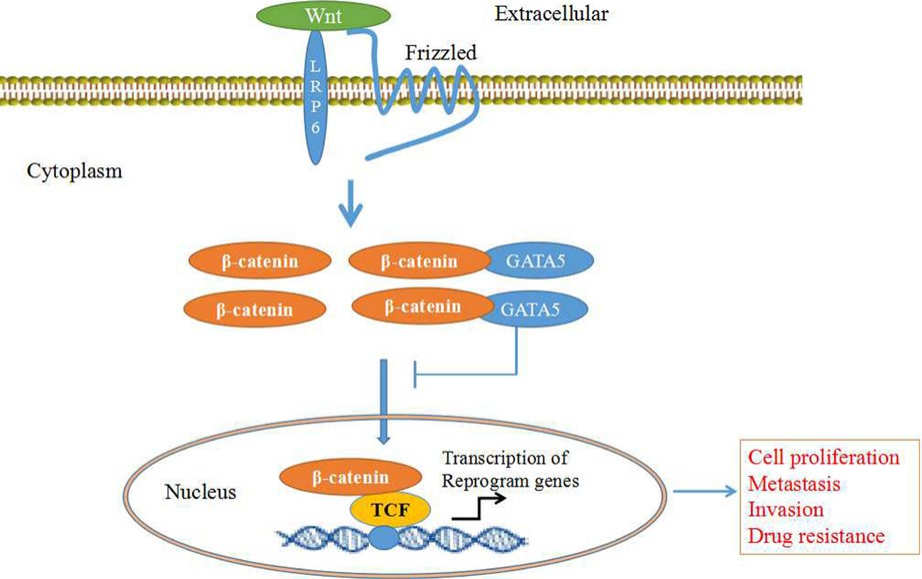
A schematic showing the role of GATA5 in suppressing the expression of reprogramming genes in HCC cells through blocking the Wnt/β‐catenin signalling. GATA5 decreases expression levels of β‐catenin and reduces its entry into the nucleus, leading to a decline in transcription of reprogramming genes which play critical roles in HCC cell proliferation, metastasis, invasion and drug resistance

Herein, we demonstrated for the first time that GATA5 was able to down‐regulate the expression of β‐catenin and the reprogramming genes p‐Oct4, Nanog, Klf4, c‐myc and EpCAM in HCC tissues and cells. To our surprise that GATA5 not only plays a role in inhibiting expression of β‐catenin, but also blocking β‐catenin enter the nucleus. GATA5 inhibits the growth and migration and invasion of HCC cells, and GATA5 synergises with paclitaxel to inhibit the growth and induce the apoptosis in HCC cells. Additionally, we revealed that the molecular mechanism of GATA5 inhibition of malignant behaviour of HCC cells occurs through the dysregulation of Wnt/β‐catenin signalling and is accompanied by the suppression of reprogramming gene expression (Figure 8). These findings suggest GATA5 application as a novel therapeutic molecule for treating HCC patients.

## 5 CONCLUSION

GATA5 harbours a function in inhibiting malignant behaviour of HCC cells, the mechanism maybe involve in dysregulation of Wnt/β‐catenin signalling and suppression of reprogramming gene expression. GATA5 is a novel target for the therapy of HCC patients.

## CONFLICTS OF INTEREST

The authors declare that they have no competing interests.

## AUTHOR CONTRIBUTION

HF, MZ, RZ, QW, W.L, YL, XD, BL and YC performed the experiments; BL, and ML analysed the clinical data and discussed the results; HF, BL, and ML drafted the manuscript, designed the experiments and revised the results; HF, RZ, QW, and JG, ML were primarily responsible for writing the manuscript. All the authors contributed to manuscript editing and approval.
